# Outcomes and short-term survivorship of fixed-bearing all-poly unicompartmental knee arthroplasty. A retrospective single-center study of 42 prostheses with a main follow-up of 32.3 months

**DOI:** 10.1007/s00402-026-06348-7

**Published:** 2026-05-20

**Authors:** Stefano Gaggiotti, Gabriel Gaggiotti, Sebastien Lustig, Cecile Batailler, Joan Carles Monllau, Santino Gaggiotti, Andreu Combalia

**Affiliations:** 1 Departament de Cirurgia i Especialitats Medicoquirúrgiques, Facultat de Medicina i Ciències de la Salut, University of Barcelona, Barcelona, Spain; 2 Orthopedic Surgery Unit, Sanatorio Mayo, Santa Fe, Argentina; 3 Department of Orthopedic Surgery and Sports Medicine, Hôpital de la Croix-Rousse, Lyon, France; 4 Institut Català de Traumatologia i Medicina de l‘Esport (ICATME), Hospital Universitario Dexeus, Barcelona, Spain

**Keywords:** Unicompartmental knee arthroplasty, UKA, All poly tibial component, AP UKA

## Abstract

**Purpose:**

All Poly (AP) tibial unicompartmental knee arthroplasty (UKA) seems to have advantages in comparison with metal-based (MB) UKA, such as greater polyethylene thickness, wear resistance, bone stock preservation and lower implant costs. The objective of this retrospective study was to evaluate the short-term clinical-functional and radiographic results, complications and survivorship of AP UKA performed consecutively with a minimum follow-up of 2 years.

**Methods:**

Retrospective cohort analysis all consecutive knees operated with medial and lateral AP UKAs, including bilateral cases, with a minimum follow-up of 2 years. Demographic data were analyzed at the patient level and clinical-functional results were assessed per knee using the KSS, KOOS and VAS scales. Radiographically, mechanical axis (HKA) correction and the presence of radiolucency > 2mm were evaluated. Complications incidence and prosthetic survivorship was determined. Pre-postoperative comparisons were performed using linear mixed-effects models with random intercept per patient to account for the non-independence of bilateral cases. Subgroup analyses involving fewer than 10 observations were considered exploratory and no formal statistical inference is drawn. A value of p < 0.05 was considered statistically significant with a confidence interval (CI) 95%.

**Results:**

42 AP UKAs were included, 5 lateral (11.9%) and 37 medial (88.1%) in 31 patients, 12 male (38.7%), 68.4 ± 8.9 years old, BMI of 32 (24.6–50.6) and mean follow-up of 32.3 months (24–44). Significant improvement in clinical KSS from 39.9 ± 3.3 to 89 ± 6.1 (Δ 49.1; 95%CI 47.2 to 51.0; p < 0.001), functional KSS from 23.3 ± 2.8 to 93.1 ± 9.3 (Δ 69.9; 95%CI 67.1 to 72.6; p < 0.001), KOOS from 33.0 ± 5.0 to 97.3 ± 6.2 (Δ 64.2; 95%CI 62.0 to 66.5; p < 0.001) and VAS from 9.4 ± 0.6 to 0.7 ± 1.3 (Δ − 8.7; 95%CI − 9.1 to − 8.3; p < 0.001) was observed in all cases. HKA for medial UKA improved from 169.6 ± 4.1 to 175.1 ± 2.5 (Δ 5.5°; 95%CI 4.6 to 6.3; p < 0.001), and for lateral UKA from 189.8 ± 4.3 to 184.6 ± 0.5 (Δ − 5.2°; 95%CI − 8.7 to − 1.7; results are descriptive only due to insufficient sample size). Six complications were observed (14.3%), all of them minor with good evolution. One case (2.4%) required TKA revision due to persistent pain after 2 years postoperatively. Prosthetic survivorship was 97.6% (95%CI 92.2%–100%).

**Conclusion:**

The AP UKA was associated with favorable clinical-functional and radiographic outcomes, few complications and prosthetic survivorship of 97.6% after an average short-term follow-up of 32.3 months (24–44) in this single-center retrospective series, which included a high proportion of cases with BMI > 30 (54.8%) and > 40 (9.7%), advanced grade 4 osteoarthritis (78.6%) and severe deformities greater than 10º (40.5%) and 15º (19%). Given the observational design, short follow-up, and absence of a control group, these findings should be interpreted with caution. Prospective comparative trials and longer-term survivorship evaluation are needed.

**Level of evidence:**

IV, retrospective cohort study.

## Introduction

Unicompartmental knee arthroplasty (UKA) is a highly effective treatment for patients with unicompartmental knee osteoarthritis [1–7]. According to recent publications and personal experience, UKA can be used in approximately 50% of knee replacements and represents an excellent alternative to total knee arthroplasty (TKA) [8–12]. It preserves healthy knee structures, proprioception and restores native biomechanics with minimal bone resection [13–17]. It is associated with less bleeding, less intraoperative, hospitalization and rehabilitation times, lower morbidity and mortality, and lower complication and infection rates, in addition to better functional results and survivorship comparable to TKA [1–10, 13–18].

There exist different tibial component types for the fixed bearing UKA: those made entirely of polyethylene—All Poly (AP), and those that have a metal base on which the polyethylene insert sits (MB). The use of AP UKA can present some advantages over modular MB, such as greater polyethylene thickness and wear resistance, elasticity modulus more similar to cement and bone, preservation of bone stock and lower implant costs [19, 20]. However, its use is not widespread at present, with MB implants being the most used [21–23]. MB implants can provide greater intraoperative flexibility and the possibility of insert replacing in case of wear [21–23]. Some biomechanical studies observed greater proximal tibial stress using AP components and better load distribution using MB implants, which may explain the higher failure rate with UKA AP reported by some studies [19, 24–27]. However, the results are not conclusive among international publications, some of which report similar functional and survival results between both types of tibial components [19, 22, 28], others better results using the AP UKA [18, 19, 29] and others with the MB UKA [30–34].

Current evidence regarding AP versus MB UKA reveals a significant divergence between national registries and specialized centers. While the Swedish Knee Arthroplasty Register reports superior long-term survivorship for AP designs [35, 36], other major databases, such as the National Joint Registry (NJR) and Australian Orthopaedic Association National Joint Replacement Registry (AOANJRR), have historically indicated higher revision rates for AP components, often associated with low-volume providers [37, 38]. Conversely, outcomes from high-volume 'Uni Users' consistently outperform national registry averages, with AP survivorship rates reaching levels comparable to TKA [39, 40]. This disparity underscores that annual surgical volume and specialized training seems to be the most critical determinants of long-term success [41, 42]. Due to the existence of inconclusive results, it represents an opportunity to provide more evidence through a study carried out in an experienced UKA center, through a current series of patients and inclusion of extended indications of UKA.

The objective of this retrospective study was to evaluate the clinical-functional and radiographic results, complications and short-term survivorship of medial and lateral AP UKAs performed consecutively by a single surgical team, with a minimum follow-up of 2 years. The hypothesis was that the use of AP tibial component for medial and lateral UKA is associated with favorable clinical-functional and radiographic results, with few complications and survivorship comparable to the international literature.

## Methods

### Level of evidence: IV, retrospective cohort study

**Fig. 1:**
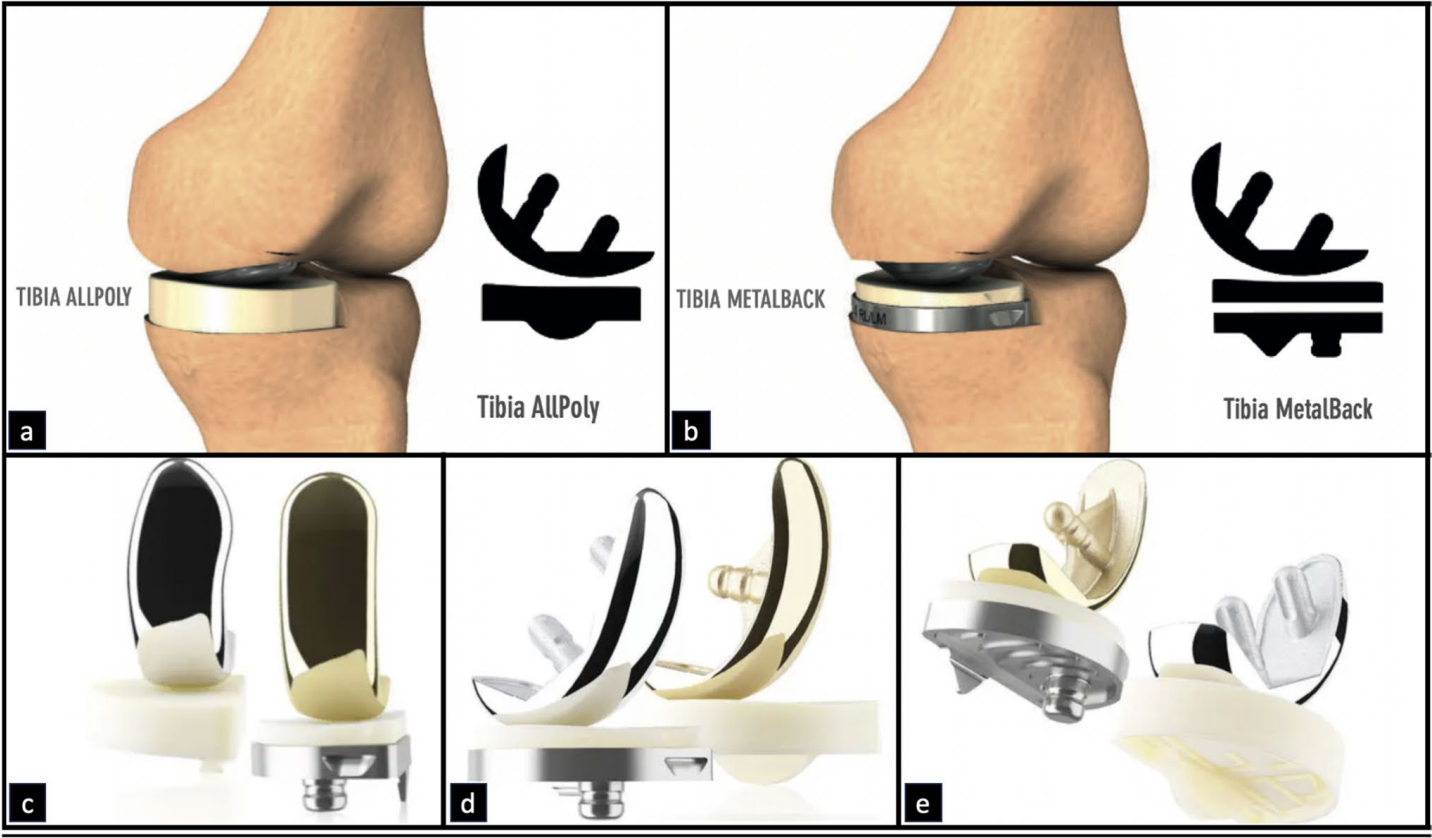
The K-mono UKA system Bioimpianti*®. a and b)* Comparative schematic representation of the AP and MB UKA. *c, d and e)* Comparative view of the AP and MB prosthetic components from the front, profile and bottom view*. UKA* Unicompartmental Knee Arthroplasty,* AP*  All-Poly*, MB*  Metal-Back

A retrospective cohort analysis of all knees operated with medial and lateral AP UKAs, including bilateral cases, implanted consecutively between November 2021 and November 2023 in a single Private Hospital, by the same surgical team (> 20 UKAs per year) and using the same surgical technique, with a minimum follow-up of 2 years was performed. Cases of UKA in which MB tibial component were used, loss to follow-up, incomplete clinical-radiographic history and patients treated with TKA or other knee surgery were excluded. In all cases, fixed bearing UKA *(K-mono system Bioimpianti®, Milan, Italy)* with 3 cut femoral resection component and ultra-high molecular weight AP tibial component (UHMWPE) were used (Fig. 1). All procedures were performed in accordance with the ethical standards of the institutional and/or national research committee, the 1964 Helsinki Declaration and its later amendments, or comparable ethical standards. The Commission Nationale Informatique & Libertés (CNIL) approved this study in Paris on April 19, 2022, under number 2226075v 0.

The medical records of all patients who met the inclusion criteria were reviewed and demographic data (age, sex and BMI) was recorded. Clinical-functional results were analyzed per knee independently using KSS, KOOS scores and degree of pain at the knee level using the VAS scale. The general knee condition was also included as a subjective punctuation between 1 to 10 (1 as the worst and 10 as the best condition), to compare in a simple way how the knee function improves after surgery as a patient’s satisfaction evaluation. The clinical-functional evaluation was done per knee both in preoperatively and in the last postoperative control, and the results were compared between them. Hospitalization time, complications incidence (including reoperation), and prosthetic survivorship at the last follow-up evaluation were analyzed. Revision was considered to be any new surgical intervention performed on the operated knee, consisting of the removal or replacement of any of the prosthetic components, and reoperation to those with preservation of the components. In case of complications, reoperation or revision, the time of evolution and its treatment were determined.

All knee radiographs were evaluated preoperatively and postoperatively using a standardized protocol based on weight-bearing antero-posterior (AP) and lateral knee radiographs, patellar axial view, and full-length standing radiographs. In addition, during the preoperative phase, all knees underwent AP valgus and varus stress radiographs. The degree and severity of osteoarthritis were assessed using the Kellgren-Lawrence classification. Mechanical limb axis (HKA) and posterior tibial slope (PTS) were measured both in the preoperative and last postoperative control, and compared between them [43]. The correction of the deformity (HKA) and the presence of radiographic changes that could compromise the implant survival (mobilization/loosening or tibial radiolucency > 2mm) were determined. The HKA angle was measured as the one created between a line that runs from the centre of the femoral head to the centre of the knee (femoral mechanical axis) and another line that runs from the centre of the knee to the centre of the ankle (tibial mechanical axis) in the full-length standing radiograph. PTS was the angle formed between the proximal epiphyseal surface of the tibia and a line following the tibial diaphyseal axis in the lateral view. All measurements were taken independently by two experienced knee surgeons, trained in UKA surgery (SG and GG). The measurement accuracy was at one decimal. Inter and intra‐observer reliability were assessed.

### Indications of the procedure

All cases after November 2021 were treated with AP UKA due to the good results published in the international literature, associated with the lower costs of the implant. Extended indications for UKA were used, as symptomatic medial or lateral unicompartmental knee osteoarthritis, regardless of patellofemoral compartment radiographic or clinical signs [3, 4, 7, 8, 12, 44]. The deformity must be correctable on stress radiographs (varus or valgus), with preservation of the contralateral femorotibial compartment. Coronal malalignment up to 20º, flexion ≥ 90º, extension deficit ≤ 15º and clinical ligamentary sufficiency in the coronal (varus and valgus stress), sagittal (Drawer and Lachman test) and rotational planes (Pivot Shift test) are mandatory. Age or BMI were not considered contraindications [3, 4, 7–9, 11, 12, 14, 15, 44].

### Surgical technique

**Fig. 2:**
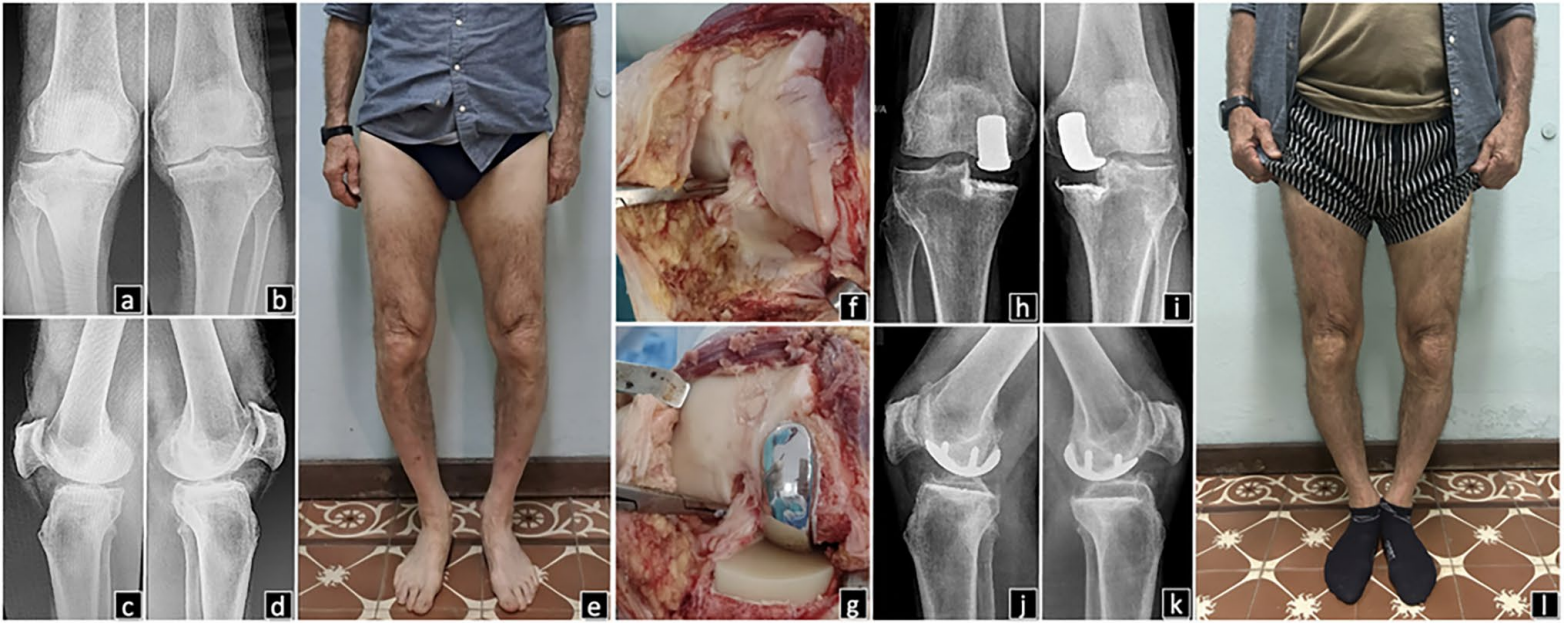
66-year-old male patient presenting with disabling bilateral knee pain that limits daily living activities. *a, b, c and d)* Preoperative anteroposterior and lateral knee radiographs. Bilateral grade 4 medial knee osteoarthritis and severe malalignment of 15º are confirmed. *e)* Varus deformity of both lower limbs. Intraoperative images of a right knee medial AP UKA. *f)* Grade 4 osteoarthritis degeneration is evident at the medial femorotibial compartment, associated with preservation of ACL and the rest of the knee compartments. *g)* Implantation of medial AP UKA as a treatment for medial femorotibial osteoarthritis. Favorable 3-year postoperative result of bilateral and simultaneous medial AP UKA. *h, i, j and k)* Postoperative anteroposterior and lateral knee radiographs showing adequate prosthetic position without signs of loosening. *l)* Restoration of the primitive limb axis after bilateral medial UKA implantation. *AP UKA* all-poly unicompartmental knee arthroplasty, *ACL* anterior cruciate ligament

In all cases functional alignment was performed, orienting the tibial component according to the Cartier angle and preserving the height of the joint line, with a posterior tibial slope up to 7º. The objective was to restore the primitive axis of the limb, avoiding overcorrection by preserving the collateral ligament integrity and obtaining a safety laxity of 2mm with the knee at 15–20º of flexion. The femoral component must be centered on the tibial component in both extension and flexion. The prosthetic components were cemented in one step, starting in all cases with the femoral component. Tibial cementation was performed by sliding the tibial component over the tibial bone surface while applying stress in valgus (for medial UKA) or varus (for lateral UKA), until it adopts its position at the location of the keel, with the knee in semiflexion of 20–30º. The knee is then brought into full extension and axial compression is applied to promote adequate cement interdigitation (Fig. 2). For unilateral cases, aspirin was used as antithrombotic prophylaxis for 30 days. In cases of bilateral UKA, dabigatran 150 mg per day was administered for 30 days.

### Rehabilitation protocol

A standardized rehabilitation protocol was used in all cases, similar for unilateral and bilateral AP UKAs. Patients were trained and instructed to perform and achieve effective isometric quadriceps contractions prior to surgery in order to facilitate a quick recovery. The early rehabilitation protocol included quadriceps, hamstrings, gastrocnemius, and soleus strengthening exercises, and ambulation with full weight-bearing allowed from the first day after surgery. Physical therapy began after 3–4 weeks and return to normal activities occurred at 6–8 weeks.

### Statistical analysis

For categorical variables, absolute frequencies and percentages were calculated. Quantitative variables were described by mean and standard deviation (SD) with confidence intervals (CI) of 95%, or median and interquartile range (IQR), depending on the distribution. For pre and postoperative comparisons, the mean differences (deltas Δ) ​​were calculated with CI 95%. Subgroup analyses with fewer than 10 observations were considered exploratory and no formal hypothesis testing was performed. Given the inclusion of 11 patients with bilateral procedures, the assumption of independence between observations was not met for standard paired tests. Therefore, all pre-postoperative comparisons for continuous variables were performed using linear mixed-effects models with a random intercept per patient. For dichotomous variables, a generalized mixed-effects model with binomial family was used. Comparisons between groups (medial vs. lateral, and across BMI categories) were also performed within the mixed-effects framework. Prosthetic survivorship was analyzed using Kaplan–Meier curves, considering prosthetic revision due to any cause. Survivorship curves were generated stratifying by compartment (medial vs lateral), including CI 95% and number of knees at risk. Given the small size of the lateral subgroup (n = 5) and the single revision event overall, survival curves are presented descriptively by compartment without formal log-rank comparison. The magnitude of clinical improvement was quantified using Cohen’s d effect size, calculated at the patient level (n = 31) to account for bilateral cases. Post hoc power estimation is not reported, as effect sizes of this magnitude render such calculations uninformative. Two experienced surgeons (SG and GG) performed the radiographic measurements two times to assess intra and inter‐observer reliability with a period of 2 months between measurements. Intraclass correlation coefficients (ICC, two-way mixed model, absolute agreement, single measures) were calculated for HKA and PTS measurements to assess inter- and intra-observer reliability. ICC values were interpreted as follows: < 0.50 = poor, 0.50–0.75 = moderate, 0.75–0.90 = good, and > 0.90 = excellent reliability. A value of p < 0.05 was considered statistically significant.

## Results

**Fig. 3:**
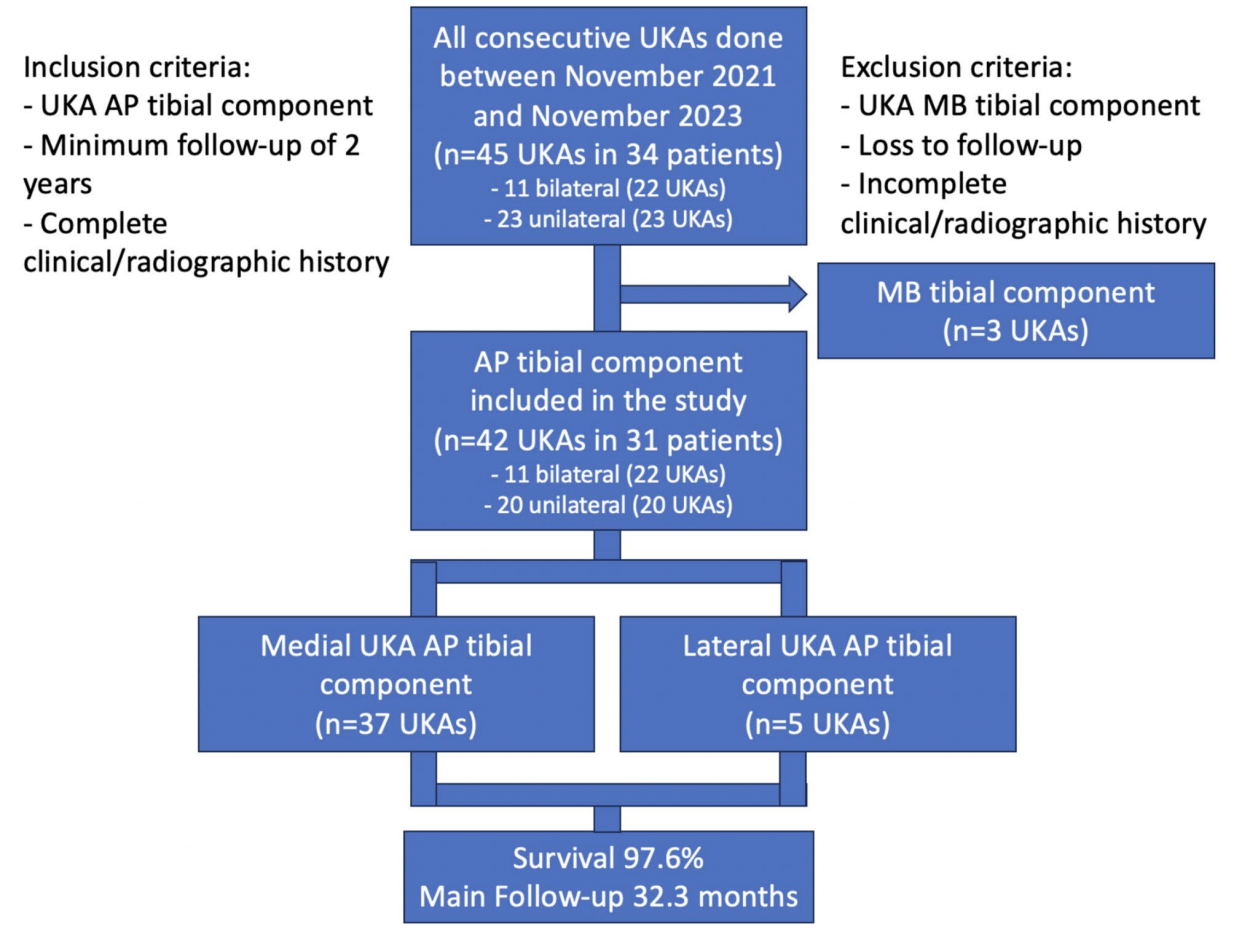
Flowchart of patients evaluated in the study after applying inclusion and exclusion criteria. *UKA AP*  All-Poly Unicompartmental Knee Arthroplasty*, UKA MB* Metal-back Unicompartmental Knee Arthroplasty

Between November 2021 and November 2023, 45 UKAs were done. Three cases were operated using MB UKA so were excluded. All AP UKA cases were evaluated after 2 years follow-up so, no cases of incomplete medical-radiographic records or loss of follow-up were observed. Finally, the series included 42 AP UKA, 5 lateral (11.9%) and 37 medial (88.1%) in 31 patients, 12 male (38.7%), with an average age of 68.4 ± 8.9 years, BMI of 32 (24.6–50.6) and average follow-up of 32.3 months (range 24–44 months). Simultaneous bilateral UKA was performed in 11 patients. In 17 patients (54.8%) the BMI was greater than 30 and in 3 (9.7%) it was greater than 40. The average hospitalization time was 29 h (24–31), 31.5 h for bilateral cases (24–48) and 27.7 h for unilateral cases (24–48) (Table 1) (Fig. 3).

**Table 1:** Demographic characteristics of the sample and analysis of clinical-functional-radiographic results

Patients	
Total	31
Age	68.4 ± 8.9
Gender	
Female	19 (61.3%)
Male	12 (38.7%)
BMI	
< 30	14 (45.2%)
30–35	10 (32.3%)
> 35	7 (22.6%)
Bilateral	11 (35.5%)
Hours of hospitalization	24.0 [24.0,34.0]
Follow-up	31.0 [25.5,38.0]
Prostheses	
Total	42
Medial	37 (88.1%)
Lateral	5 (11.9%)
KL	
3	9 (21.4%)
4	33 (78.6%)
Malalignment > 10º preop	17 (40.5%)
Coronal subluxation	14 (33.3%)

Variable	Mean ± SD PRE	Mean ± SD POP	Δ (IC95%)	P value
Clinical KSS	39.9 ± 3.3	89.0 ± 6.1	+ 49.1 (47.2 to 51.0)	< 0.001
Functional KSS	23.3 ± 2.8	93.1 ± 9.3	+ 69.9 (67.1 to 72.6)	< 0.001
KOOS	33.0 ± 5.0	97.3 ± 6.2	+ 64.2 (62.0 to 66.5)	< 0.001
VAS	9.4 ± 0.6	0.7 ± 1.3	− 8.7 (− 9.1 to − 8.3)	< 0.001
General Condition	1.9 ± 1.5	8.9 ± 0.9	+ 7.0 (6.5 to 7.6)	** < 0.001**
Extension	3.9 ± 3.2	0.9 ± 1.6	− 3.0 (− 3.6 to − 2.4)	** < 0.001**
Flexion	104.8 ± 5.6	120.1 ± 5.1	+ 15.4 (13.4 to 17.3)	** < 0.001**
HKA	172.0 ± 7.8	176.2 ± 3.9	+ 4.2 (2.7 to 5.7)	** < 0.001**
HKA varus	169.6 ± 4.1	175.1 ± 2.5	+ 5.5 (4.6 to 6.3)	** < 0.001**
HKA valgus	189.8 ± 4.3	184.6 ± 0.5	-5.2 (-8.7 to -1.7)	-
PTS	4.4 ± 1.5	4.2 ± 1.3	− 0.1 (− 0.6 to 0.4)	**0.638**

Pre-postoperative comparisons performed using linear mixed-effects models with random intercept per patient. HKA varus = medial UKA (n = 37); HKA valgus = lateral UKA (n = 5, descriptive only — no formal statistical testing performed due to insufficient sample size)

*BMI* body mass index, *KL* kellgren-lawrence, *PRE* preoperative, *POP* postoperative, *KSS* knee score system, *KOOS* knee osteoarthritis outcome score, *VAS* visual analog scale, *HKA* hip-knee-ankle angle, *PTS* posterior tibial slope

**Fig. 4:**
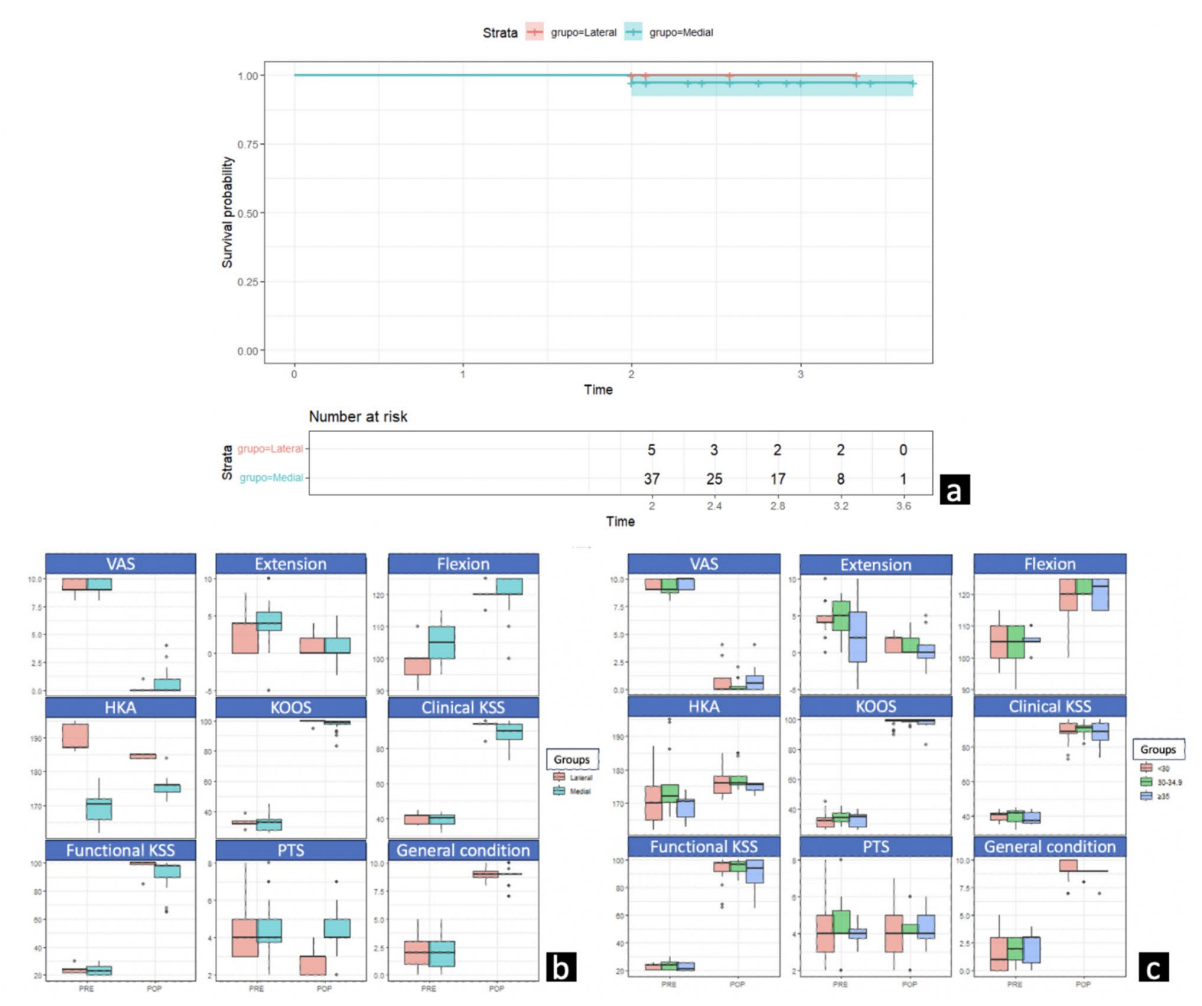
Survival analysis using Kaplan–Meier curve and graphical outcomes representation according to medial–lateral AP UKA and BMI groups. *a)* Kaplan–Meier revision-free survival curve stratified by compartment (medial n = 37; lateral n = 5), with 95% confidence intervals and number of knees at risk at each time point. Given the small lateral subgroup, curves are presented descriptively without formal log-rank comparison. *b and c)* Box plots showing pre- and postoperative distribution of clinical, functional, and radiographic outcomes stratified by compartment and BMI category. Lateral subgroup data (n = 5) are shown for descriptive purposes only. AP *UKA*  All-Poly Unicompartmental Knee Arthroplasty, *BMI* Body Mass Index,* PRE* preoperative* POP* postoperative* KSS* Knee Score System*, KOOS* Knee Osteoarthritis Outcome Score*, VAS* Visual Analog Scale*, HKA* Hip-Knee-Ankle angle*, PTS* Posterior Tibial Slope

A significant improvement was observed in all clinical-functional parameters at the last postoperative control after an average follow-up of 32.3 months (range 24–44 months). Clinical KSS went from 39.9 ± 3.3 to 89.0 ± 6.1 (Δ 49.1; 95%CI 47.2 to 51.0; p < 0.001), functional KSS from 23.3 ± 2.8 to 93.1 ± 9.3 (Δ 69.9; 95%CI 67.1 to 72.6; p < 0.001) and KOOS from 33.0 ± 5.0 to 97.3 ± 6.2 (Δ 64.2; 95%CI 62.0 to 66.5; p < 0.001). The general condition of the knee using a scale from 1 to 10, during the preoperative period was on average 1.9 ± 1.5 with a VAS pain grade of 9.4 ± 0.6, which improved significantly to 8.9 ± 0.9, with a VAS pain grade of 0.7 ± 1.3 (Δ 7.0; 95%CI 6.5 to 7.6; p < 0.001) and (Δ − 8.7; 95%CI − 9.1 to − 8.3; p < 0.001) respectively. Regarding the range of motion, the average preoperative flexion was 104.8 ± 5.6 and extension was 3.9 ± 3.2, which improved to 120.1 ± 5.1 and 0.9 ± 1.6 (Δ 15.4; 95%CI 13.4 to 17.3; p < 0.001) and (Δ − 3.0; 95%CI − 3.6 to − 2.4; p < 0.001) respectively. No statistically significant differences were observed in clinical-functional scores or clinical parameters between medial and lateral UKA, or between the different BMI categories (Table 2) (Fig. 4).

**Table 2:** Comparative analysis of clinical-functional and radiographic results evaluated between compartments (medial n = 37 vs lateral n = 5) and BMI categories, all at the knee level (n = 42)

	Medial (N = 37)	Lateral (N = 5)	p-value	Total (N = 42)
Age				
Mean (SD)	67.4 (9.75)	71.2 (5.26)	0.471	67.9 (9.36)
Median [Q1, Q3]	70.0 [62.0, 76.0]	72.0 [67.0, 74.0]		70.0 [63.5, 75.5]
BMI				
Mean (SD)	32.1 (5.91)	29.2 (3.43)	0.303	31.8 (5.72)
Median [Q1, Q3]	29.1 [27.6, 34.8]	30.6 [26.6, 31.7]		29.6 [27.6, 34.8]
Clinical KSS PRE				
Mean (SD)	39.8 (3.27)	40.4 (3.78)	0.814	39.9 (3.29)
Median [Q1, Q3]	40.0 [37.0, 42.0]	42.0 [37.0, 42.0]		40.5 [37.0, 42.0]
Functional KSS PRE				
Mean (SD)	23.2 (2.73)	24.2 (3.49)	0.552	23.3 (2.80)
Median [Q1, Q3]	24.0 [20.0, 26.0]	24.0 [22.0, 24.0]		24.0 [21.0, 26.0]
Clinical KSS POP				
Mean (SD)	88.5 (6.17)	92.4 (4.77)	0.122	89.0 (6.10)
Median [Q1, Q3]	90.0 [85.5, 94.0]	94.0 [94.0, 94.0]		90.0 [86.0, 94.0]
Functional KSS POP				
Mean (SD)	92.6 (9.56)	96.8 (6.61)	0.088	93.1 (9.29)
Median [Q1, Q3]	98.0 [90.0, 98.0]	100 [99.0, 100]		98.0 [90.0, 99.0]
Flexion PRE				
Mean (SD)	106 (4.97)	99.0 (7.42)	0.039	105 (5.63)
Median [Q1, Q3]	105 [100, 110]	100 [95.0, 100]		105 [100, 110]
Extension PRE				
Mean (SD)	4.03 (3.24)	3.20 (3.35)	0.440	3.93 (3.23)
Median [Q1, Q3]	4.00 [3.00, 5.00]	4.00 [0, 4.00]		4.00 [3.00, 5.00]
Flexion POP				
Mean (SD)	120 (5.33)	120 (3.54)	0.71	120 (5.12)
Median [Q1, Q3]	120 [120, 125]	120 [120, 120]		120 [120, 125]
Extension POP				
Mean (SD)	0.892 (1.65)	1.20 (1.79)	0.849	0.929 (1.64)
Median [Q1, Q3]	0 [0, 2.00]	0 [0, 2.00]		0 [0, 2.00]
VAS PRE				
Mean (SD)	9.41 (0.599)	9.20 (0.837)	0.587	9.38 (0.623)
Median [Q1, Q3]	9.00 [9.00, 10.0]	9.00 [9.00, 10.0]		9.00 [9.00, 10.0]
VAS POP				
Mean (SD)	0.784 (1.40)	0.200 (0.447)	0.374	0.714 (1.33)
Median [Q1, Q3]	0 [0, 1.00]	0 [0, 0]		0 [0, 1.00]
KOOS PRE				
Mean (SD)	33.1 (5.13)	33.0 (4.00)	0.969	33.0 (4.97)
Median [Q1, Q3]	33.0 [28.0, 35.0]	32.0 [32.0, 34.0]		32.5 [28.0, 35.0]
KOOS POP				
Mean (SD)	97.0 (6.55)	99.0 (2.24)	0.16	97.3 (6.21)
Median [Q1, Q3]	99.0 [98.0, 100]	100 [100, 100]		99.0 [98.0, 100]
General Condition PRE				
Mean (SD)	1.78 (1.46)	2.20 (1.92)	0.676	1.83 (1.50)
Median [Q1, Q3]	2.00 [0, 3.00]	2.00 [1.00, 3.00]		2.00 [0.250, 3.00]
General Condition POP				
Mean (SD)	8.85 (0.917)	9.00 (0.816)	0.898	8.86 (0.899)
Median [Q1, Q3]	9.00 [9.00, 9.00]	9.00 [8.75, 9.25]		9.00 [9.00, 9.00]
HKA PRE				
Mean (SD)	170 (4.08)	190 (4.32)	< 0.001	172 (7.76)
Median [Q1, Q3]	171 [166, 172]	187 [187, 194]		171 [167, 174]
HKA POP				
Mean (SD)	175 (2.47)	185 (0.548)	< 0.001	176 (3.89)
Median [Q1, Q3]	176 [174, 176]	185 [184, 185]		176 [174, 177]
PTS PRE				
Mean (SD)	4.32 (1.43)	4.60 (2.07)	0.984	4.36 (1.50)
Median [Q1, Q3]	4.00 [4.00, 5.00]	4.00 [3.00, 5.00]		4.00 [3.25, 5.00]
PTS POP				
Mean (SD)	4.43 (1.19)	2.80 (0.837)	0.00557	4.24 (1.27)
Median [Q1, Q3]	4.00 [4.00, 5.00]	3.00 [2.00, 3.00]		4.00 [3.25, 5.00]
KL				
Mean (SD)	3.76 (0.435)	4.00 (0)	0.219	3.79 (0.415)
Median [Q1, Q3]	4.00 [4.00, 4.00]	4.00 [4.00, 4.00]		4.00 [4.00, 4.00]
Subluxation PRE				
No	24 (64.9)	4 (80.0)	0.65	28 (66.7)
Yes	13 (35.1)	1 (20.0)		14 (33.3)
Hours of hospitalization				
Mean (SD)	29.4 (6.78)	32.0 (10.2)	0.487	29.7 (7.16)
Median [Q1, Q3]	24.0 [24.0, 34.0]	28.0 [24.0, 36.0]		24.0 [24.0, 34.0]
Evolution				
Minor Complications	5 (13.5)	1 (20.0)	0.619	6 (14.3)
No complications	31 (83.8)	4 (80.0)		35 (83.3)
Revision	1 (2.7)	0 (0)		1 (2.4)

	< 30 (N = 21)	30–34.9 (N = 12)	≥ 35 (N = 9)	p-value	Total (N = 42)
Age					
Mean (SD)	68.8 (9.63)	70.8 (6.10)	61.9 (10.5)	0.127	67.9 (9.36)
Median [Q1, Q3]	71.0 [65.0, 76.0]	69.5 [65.8, 77.3]	62.0 [54.0, 70.0]		70.0 [63.5, 75.5]
BMI					
Mean (SD)	27.4 (1.30)	32.7 (1.60)	40.6 (4.72)	< 0.001	31.8 (5.72)
Median [Q1, Q3]	27.6 [26.6, 28.7]	32.6 [31.6, 34.1]	37.9 [37.8, 43.5]		29.6 [27.6, 34.8]
Clinical KSS PRE					
Mean (SD)	40.2 (2.68)	39.9 (4.27)	39.0 (3.35)	0.64	39.9 (3.29)
Median [Q1, Q3]	41.0 [38.0, 42.0]	42.0 [36.8, 43.3]	38.0 [36.0, 42.0]		40.5 [37.0, 42.0]
Functional KSS PRE					
Mean (SD)	22.9 (2.19)	24.3 (3.68)	22.8 (2.73)	0.526	23.3 (2.80)
Median [Q1, Q3]	24.0 [21.0, 24.0]	24.0 [21.0, 26.5]	22.0 [20.0, 26.0]		24.0 [21.0, 26.0]
Clinical KSS POP					
Mean (SD)	88.6 (6.52)	90.2 (4.22)	88.0 (7.71)	0.898	89.0 (6.10)
Median [Q1, Q3]	89.0 [88.0, 94.0]	91.0 [89.0, 92.5]	89.0 [84.0, 94.0]		90.0 [86.0, 94.0]
Functional KSS POP					
Mean (SD)	93.3 (9.87)	95.0 (5.10)	89.9 (12.5)	0.906	93.1 (9.29)
Median [Q1, Q3]	98.0 [92.0, 98.0]	97.0 [92.0, 99.3]	94.0 [83.5, 100]		98.0 [90.0, 99.0]
Flexion PRE					
Mean (SD)	105 (6.31)	105 (6.20)	106 (3.00)	0.809	105 (5.63)
Median [Q1, Q3]	105 [100, 110]	105 [100, 110]	105 [105, 105]		105 [100, 110]
Extension PRE					
Mean (SD)	4.38 (2.11)	4.33 (2.90)	2.33 (5.20)	0.599	3.93 (3.23)
Median [Q1, Q3]	4.00 [4.00, 5.00]	5.00 [3.00, 7.00]	4.00 [0, 5.00]		4.00 [3.00, 5.00]
Flexion POP					
Mean (SD)	119 (6.05)	122 (2.57)	120 (5.00)	0.325	120 (5.12)
Median [Q1, Q3]	120 [115, 125]	120 [120, 125]	120 [115, 125]		120 [120, 125]
Extension POP					
Mean (SD)	1.24 (1.18)	0.833 (1.34)	0.333 (2.69)	0.24	0.929 (1.64)
Median [Q1, Q3]	2.00 [0, 2.00]	0 [0, 2.00]	0 [0, 0]		0 [0, 2.00]
VAS PRE					
Mean (SD)	9.43 (0.507)	9.17 (0.835)	9.56 (0.527)	0.502	9.38 (0.623)
Median [Q1, Q3]	9.00 [9.00, 10.0]	9.00 [8.75, 10.0]	10.0 [9.00, 10.0]		9.00 [9.00, 10.0]
VAS POP					
Mean (SD)	0.571 (1.08)	0.333 (0.651)	1.56 (2.13)	0.22	0.714 (1.33)
Median [Q1, Q3]	0 [0, 1.00]	0 [0, 0.250]	1.00 [0, 2.00]		0 [0, 1.00]
KOOS PRE					
Mean (SD)	32.3 (5.13)	34.7 (4.23)	32.7 (5.52)	0.299	33.0 (4.97)
Median [Q1, Q3]	32.0 [28.0, 34.0]	34.5 [31.8, 37.5]	35.0 [28.0, 35.0]		32.5 [28.0, 35.0]
KOOS POP					
Mean (SD)	98.2 (2.87)	98.8 (1.66)	93.1 (12.2)	0.702	97.3 (6.21)
Median [Q1, Q3]	99.0 [99.0, 100]	99.0 [98.8, 100]	98.0 [96.0, 100]		99.0 [98.0, 100]
General Condition PRE					
Mean (SD)	1.71 (1.71)	2.00 (1.04)	1.89 (1.62)	0.716	1.83 (1.50)
Median [Q1, Q3]	1.00 [0, 3.00]	2.00 [1.00, 3.00]	3.00 [0, 3.00]		2.00 [0.250, 3.00]
General Condition POP					
Mean (SD)	9.07 (0.926)	8.80 (0.422)	8.44 (1.13)	0.131	8.86 (0.899)
Median [Q1, Q3]	9.00 [9.00, 10.0]	9.00 [9.00, 9.00]	9.00 [9.00, 9.00]		9.00 [9.00, 9.00]
HKA PRE					
Mean (SD)	171 (7.10)	176 (10.1)	169 (3.61)	0.35	172 (7.76)
Median [Q1, Q3]	170 [165, 175]	172 [170, 176]	171 [166, 172]		171 [167, 174]
HKA POP					
Mean (SD)	176 (4.13)	178 (4.31)	175 (1.67)	0.255	176 (3.89)
Median [Q1, Q3]	176 [173, 178]	176 [176, 178]	175 [174, 176]		176 [174, 177]
PTS PRE					
Mean (SD)	4.43 (1.66)	4.50 (1.68)	4.00 (0.707)	0.76	4.36 (1.50)
Median [Q1, Q3]	4.00 [3.00, 5.00]	4.00 [4.00, 5.25]	4.00 [4.00, 4.00]		4.00 [3.25, 5.00]
PTS POP					
Mean (SD)	4.29 (1.38)	4.17 (1.34)	4.22 (0.972)	0.998	4.24 (1.27)
Median [Q1, Q3]	4.00 [3.00, 5.00]	4.00 [4.00, 4.50]	4.00 [4.00, 5.00]		4.00 [3.25, 5.00]
KL					
Mean (SD)	3.86 (0.359)	3.75 (0.452)	3.67 (0.500)	0.484	3.79 (0.415)
Median [Q1, Q3]	4.00 [4.00, 4.00]	4.00 [3.75, 4.00]	4.00 [3.00, 4.00]		4.00 [4.00, 4.00]
Subluxation PRE					
No	13 (61.9)	8 (66.7)	7 (77.8)	0.837	28 (66.7)
Yes	8 (38.1)	4 (33.3)	2 (22.2)		14 (33.3)
Hours of hospitalization					
Mean (SD)	29.9 (7.84)	29.0 (7.41)	30.0 (5.74)	0.715	29.7 (7.16)
Median [Q1, Q3]	24.0 [24.0, 36.0]	24.0 [24.0, 32.5]	34.0 [24.0, 34.0]		24.0 [24.0, 34.0]
Evolution					
Minor Complications	6 (28.6)	0 (0)	0 (0)	0.0217	6 (14.3)
No complications	15 (71.4)	12 (100)	8 (88.9)		35 (83.3)
Revision	0 (0)	0 (0)	1 (11.1)		1 (2.4)

Lateral subgroup comparisons (n = 5) are exploratory only; p-values are shown for reference but the test is underpowered and no formal inference should be drawn All comparisons performed using linear mixed-effects models with random intercept per patient

*PRE* preoperative, *POP* postoperative, *BMI* body mass index, *KSS* knee score system, *KOOS* knee osteoarthritis outcome score, *VAS* visual analog scale, *HKA* hip-knee-ankle angle, *PTS* posterior tibial slope, *KL* kellgren-lawrence

Radiographically, 9 cases (21.4%) presented grade 3 and 33 (78.6%) grade 4 osteoarthritis according to KL. For medial UKAs (88.1%), HKA was corrected in the postoperative evaluation from 169.6 ± 4.1 to 175.1 ± 2.5 (Δ 5.5°; 95%CI 4.6 to 6.3; p < 0.001), and for lateral UKAs (11.9%), from 189.8 ± 4.3 to 184.6 ± 0.5 (Δ − 5.2°; 95%CI − 8.7 to − 1.7; given the small sample size, results are presented descriptively without formal statistical testing). In 13 cases of medial UKA (35.1%) and 1 case of lateral UKA (20%), coronal subluxation was observed in the preoperative evaluation, which was corrected after UKA implantation. In 15 cases of medial UKA (40.5%) and 2 cases of lateral UKA (40%), the preoperative deformity was severe > 10º. No statistically significant differences were observed between pre and postoperative PTS, with values ​​of 4.4 ± 1.5 and 4.2 ± 1.3 respectively (Δ − 0.1°; 95%CI − 0.6 to 0.4; p = 0.495). Inter-observer reliability was excellent for HKA (ICC = 0.94; 95%CI 0.89–0.97; p < 0.001) and good for PTS (ICC = 0.81; 95%CI 0.72–0.88; p < 0.001). Intra-observer reliability was good for HKA (ICC = 0.87; 95%CI 0.79–0.92; p < 0.001) and good for PTS (ICC = 0.83; 95%CI 0.74–0.89; p < 0.001). No statistically significant differences were observed in radiographic measurements between medial and lateral UKA, or between the different BMI categories. One case of overcorrection was observed, with a preoperative HKA of 176º and postoperative HKA of 184º, without impact on the clinical results after 19 months of follow-up. No implant mobilization, loosening or radiolucency > 2mm were observed (Table 2) (Fig. 4).

Six complications (14.3%) were observed, all of them minor that did not require revision surgery and evolved favorably. One case (2.4%) presented superficial phlebitis with negative Doppler at the third postoperative week, treated with aspirin and rest. Three cases (7.1%) presented patellar bursitis after 3 and 4 months postoperatively, which required puncture drainage. One case (2.4%) presented superficial wound necrosis that required reoperation with debridement and surgical closure 3 months postoperatively. In one case (2.4%) there was an intraoperatively medial collateral ligament (MCL) injury that required its suture, with limb axis overcorrection without clinical repercussions. A female patient with BMI 37.9 developed persistent mechanical pain at 3 months postoperatively. Conservative measures such as rest, analgesia and infiltrations were applied. Due to symptomatic persistence, revision of UKA to TKA was performed 2 years postoperatively. The revision rate was 2.4%, with a prosthetic survivorship of 97.6% after an average follow-up of 32.3 months (range 24—44 months). Revision-free survivorship at 2 years was 97.3% (95%CI 92.2%–100%) in the medial group, which corresponds to a cumulative incidence of revision of 2.7% in that period; there were no revisions in the lateral group until 2 years. Given the small size of the lateral subgroup (n = 5 knees) and the absence of any revision event in that group, formal log-rank comparison between compartments was not performed; survival curves are presented descriptively (Fig. 4). No statistically significant relationship was observed between complications and sex (p = 1), age (p = 0.68) or BMI (p = 0.09).

The magnitude of clinical improvement was quantified at the patient level (n = 31) to account for intra-subject correlation in bilateral cases. The mean pre-postoperative difference in clinical KSS was 48.75 points (SD 6.76), corresponding to a very large effect size (Cohen’s d = 7.2), indicating a clinically meaningful and consistent improvement across the cohort. Post hoc power estimation is not reported separately, as effect sizes of this magnitude render such calculations uninformative.

## Discussion

The most important findings of the present retrospective study were that the AP UKA presented favorable clinical-functional and radiographic results, few complications and prosthetic survivorship of 97.6% after an average of 32.3 months (range 24—44 months) of follow-up. These results were obtained in this series composed by a high percentage of cases with BMI > 30 (54.8%), advanced grade 4 osteoarthritis (78.6%), subluxation in the coronal plane (33.3%) and severe malalignment > 10º (40.5%) and > 15º (19%).

This consecutive series of 42 medial and lateral AP UKAs with a minimum follow-up of 2 years showed a significant improvement in clinical-functional results and high levels of satisfaction in the last postoperative control, similar to that reported by the international literature [18, 45–48]. Although it was not evaluated in this study, when analyzing both types of tibial components in UKA (AP and MB), some authors observed better results by using AP components [18, 19, 29], while others reported higher values ​​with MB [30–32]. In their study of 104 UKAs with 10 years of follow-up, Lee et al. did not observe significant differences in functional results between AP and MB UKA [28]. Similarly, the meta-analysis conducted by Gianluca Costa et al. did not show significant differences in terms of functional results between these two types of implants [19, 22, 28]. As Cerciello et al. pointed out recently in his current concepts paper, MB implants although more common than AP implants do not have clearly superior outcomes [33].

In the present series, more than half of the patients registered a BMI > 30 (54.8%). There are reports and meta-analyses that support the use of UKA in obese patients, with good long-term results [7, 19, 45, 49–52]. In their comparative study, Foo et al. observed no significant differences in the functional results of obese patients (BMI > 30) treated with AP vs. MB tibial component UKA [19]. However, when performing a subanalysis in patients with BMI > 35, they observed worse results in AP medial UKA, with similar prosthetic survival at 2 years postoperatively (AP: 98.7% and MB: 99.4%) [19]. In a recent retrospective propensity-matched analysis published in 2024, when comparing cases of AP and MB medial UKA in obese people (BMI > 30), De Bernardinis et al. observed significantly better functional results in the AP group after a minimum follow-up of 5 years and 100% survival [24]. In this series, age was not considered as a contraindication for the procedure either, since it represents a therapeutic alternative that benefits all age groups [7, 45, 46]. The AP UKA seems to be especially useful in elderly patients with osteoporotic bone, with polyethylene having an elasticity modulus closer to cement and bone, as reported by Bruce et al., observing greater survival with AP UKA in patients > 70 years than for those under 70 years old [46]. Additionally, in young patients the AP tibial component provides a greater thickness of polyethylene, favoring prolonged survival [18].

AP UKA designs eliminate the risk of polyethylene insert dislocation by presenting a single interface [46]. Polyethylene wear is cited as one of the UKAs failure mechanisms, especially in old series [53, 54]. Currently, due to the use of UHMWPE and changes in sterilization technique, this complication is less frequent [46]. The use of AP UKA provides a greater thickness of polyethylene for the same height of tibial component, reducing the incidence of this complication and favoring bone preservation [20, 46]. In this series, no cases of polyethylene wear were observed after a mean follow up of 32.3 months (range 24–44 months). Lustig et al. mentions the absence of clinical or radiographic wear after a maximum follow-up of 13 years [45]. In the series by Bruce et al., after a minimum follow-up of 10 years, they observed 3 cases of revision secondary to polyethylene wear, which represents 1.4% of the total [46].

The use of an AP component, whether flat or with a single keel centralized on the tibial surface, reduces the risk of tibial plateau fracture, since it stabilizes the implant without weakening the peripheral area of ​​the bone surface with fixation holes, as occurs with MB components [55]. In this series, no complications due to tibial plateau fracture were observed, nor in the majority of series published in the literature [18, 29, 45]. Another surgical gesture associated with reducing the risk of tibial plateau fracture is the alignment of the tibial component with respect to the Cartier angle, which reduces stress on the tibial cortical bone [5, 6, 14, 15, 56]. Due to the absence of fixation holes and less bone resection, the use of AP components is associated with less loss of tibial bone stock and less frequent requirement for stems in case of revision to TKA, compared to MB implants [20, 57].

One case (2.4%), a female patient with a BMI of 37.9, due to persistent medial mechanical pain without radiographic signs of prosthetic loosening, required revision of medial UKA to TKA 2 years postoperatively. Prosthetic survivorship was 97.6% after an average follow-up of 32.3 months (range 24–44 months), similar to other reports in the international literature [18, 24, 28, 31, 45, 47, 48]. Despite the possible advantages of the AP tibial component over MB, several biomechanical studies observed greater stress on the proximal tibia in the AP components that can lead to unexplained residual pain due to a lower load distribution over the tibial surface in comparison with the MB design [19, 24–27]. However, the most frequently reported cause of revision in the literature is aseptic loosening of the tibial component, with no significant differences between AP or MB models [22, 31, 58]. Sessa et al. in a long-term comparative study, found a significative higher survivorship for AP UKA (97.6%) vs MB UKA (89.5%) after a mean follow-up of 11.5 years, similar to Cartier’s series after 15 years follow-up [18, 34]. In the meta-analysis carried out in 2020 by Gianluca Costa et al. which included 9 studies with a minimum follow-up of 2 years, 1101 UKAs and 87 revisions, they observed similar survival between implants, and higher but not statistically significative failure risk in female patients treated with AP components [22]. Recently in 2025, Pousalehian et al. in their meta-analysis of 35,639 knees and 18 studies, they observed that revision rates at 2, 5, and 10 years were lower for the MB group, with the main failure cause being tibial loosening [23]. According to Cerciello et al., MB implants although more common than AP implants do not have clearly superior outcomes [33].

The current study has some potential limitations. The series originates from a single-surgeon institution. Other limitations were small sample size, especially for lateral UKA, short follow-up duration for survivorship interpretation, lack of comparative MB control group, small subgroup sizes, potential selection bias inherent to retrospective consecutive series and bilateral cases that potentially can influence independence of observations. Comparative studies consisting of a larger number of patients and longer follow-up are necessary to obtain more robust conclusions. The minimum 2-year follow-up, the inclusion of medial and lateral UKA, the high proportion of extreme cases, and the contemporaneity of the cohort stand out, since the majority of the reports in the literature are made up of historical series. Despite that, findings are limited to a selected cohort in a high-volume center and may not be generalizable.

## Conclusion

In the present retrospective cohort, the UKA with AP tibial component presented favorable clinical-functional and radiographic results, few complications and prosthetic survivorship of 97.6% after an average short-term follow-up of 32.3 months (range 24–44 months) in this series with a high proportion of cases with BMI > 30 (54.8%) and > 40 (9.7%), advanced grade 4 osteoarthritis (78.6%) and severe deformities greater than 10º (40.5%) and 15º (19%). The use of the AP tibial component represents a valid alternative to the MB design, the most used worldwide. Despite that, prospective comparative trials and longer-term survivorship evaluation are needed.

## Data Availability

No datasets were generated or analysed during the current study.
